# Supplementary material for: Retinoic acid signaling regulates spatiotemporal specification of human green and red cones
Source: PLoS Biol. 2024 Jan 11;22(1):e3002464. doi: 10.1371/journal.pbio.3002464 (PMC10783767; doi:10.1371/journal.pbio.3002464)
Supplement: S1 Fig — (A) Identification of HEK293 cells using Hoechst (light gray) in the experiment in Fig 1I and 1J. M-opsin (blue) and L-opsin (pink). Blue arrow indicates a cell expressing M-opsin mRNA only. Pink arrows indicate cells expressing L-opsin mRNA only. Purple arrow indicates a cell expressing both M-opsin mRNA and L-opsin mRNA. Black arrow indicates an untransfected cell. Note that the Hoechst signal is reduced in cells that express M-opsin mRNA and/or L-opsin mRNA, likely due to the colorimetric signal blocking the fluorescent signal. (B) Identification of retinal layers using Hoechst (light gray) in the experiment in Fig 1K and 1L. M-opsin (blue) and L-opsin (pink). No cones co-expressed M-opsin mRNA and L-opsin mRNA. ONL, outer nuclear layer; OPL, outer plexiform layer; INL, inner nuclear layer. (C) Quantification of M-opsin mRNA-expressing, L-opsin mRNA-expressing, or M+L-opsin mRNA-expressing cells that express M/L-opsin protein for the experiments conducted in HEK293 cells in Fig 1I and 1J and adult human retina in Fig 1K and 1L. (PDF) [file pbio.3002464.s001.pdf]

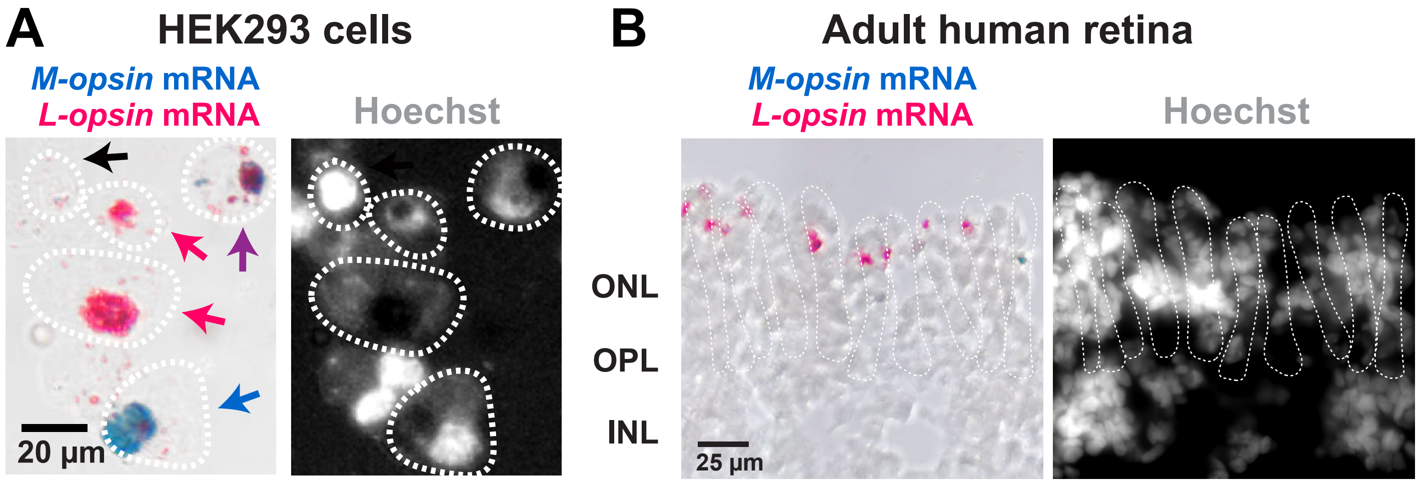


C

| | Adult human retina | | | HEK293 | | |
|---|---|---|---|---|---|---|
| | Total | M/L-opsin protein + | M/L-opsin protein - | Total | M/L-opsin protein + | M/L-opsin protein - |
| *L-opsin* mRNA + cells | 301 | 294 | 7 | 50 | 48 | 2 |
| *M-opsin* mRNA + cells | 83 | 81 | 2 | 58 | 58 | 0 |
| *M- and L-opsin* mRNA + cells | 0 | 0 | 0 | 128 | 124 | 4 |

**Supplemental Figure 1**
