# Supplementary figures and images for: Retinoic acid signaling regulates spatiotemporal specification of human green and red cones
Source: PLoS Biol. 2024 Jan 11;22(1):e3002464. doi: 10.1371/journal.pbio.3002464 (PMC10783767; doi:10.1371/journal.pbio.3002464)

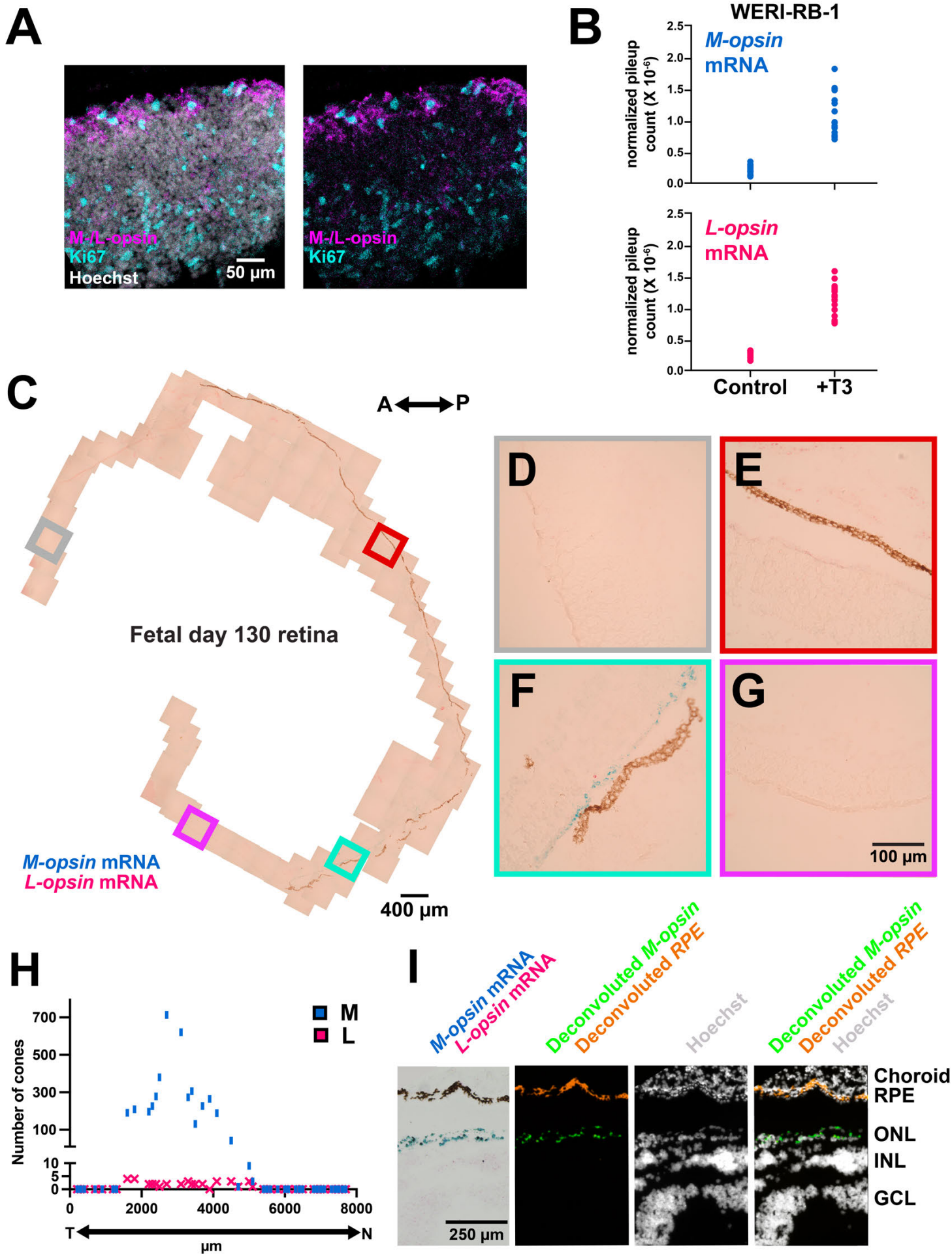

Supplemental Figure 2

Supplement: S2 Fig — (A) 20 μm section of a fetal day 122 retina with expression of M-/L-opsin protein (magenta), Ki67 (cyan), and Hoechst/nuclei (white). M-/L-opsin protein is observed in the ONL. Ki67 expression indicates proliferating cells. (B) M- and L-opsin expression in control- and T3-treated WERI-Rb-1 retinoblastoma cells (N = 1 experiment). The WERI-Rb-1 retinoblastoma cell line expresses M- and L-opsin at low levels [50]. T3, the active form of thyroid hormone, induces M- and L-opsin expression in WERI-Rb-1 cells [15,51]. Values indicate total pileup count normalized to total read count. Each data point indicates 1 nucleotide difference in M- or L-opsin. Original data sets are in S5 Data. (C–I) Analysis of M- and L-opsin mRNA expression in 130-day-old human fetal retina. (C) Example of M- and L-opsin mRNA expression in 130-day-old human fetal retina section. Anterior = left, posterior = right. Dorsal/ventral orientation is unknown. Colored boxes indicate regions shown in (D–G) zoomed in regions from (C). The central region in (F) expressed opsin mRNA. The more peripheral regions in (D), (E), and (G) show no expression of opsin mRNA. (H) Quantification of cones expressing M- and L-opsin mRNA from the temporal to nasal sides of the globe. (I) Identification of retinal layers using Hoechst (light gray) in the experiment in (S2C–S2H Fig). M-opsin (blue), L-opsin (pink), and retinal pigmented epithelial (RPE, dark brown) colorimetric signals were deconvoluted to generate pseudo fluorescent images. These were overlaid with Hoechst nuclear counterstains to visualize retinal layers. ONL, outer nuclear layer; OPL, outer plexiform layer; INL, inner nuclear layer. Original data sets are in S5 Data. (PDF) [file pbio.3002464.s002.pdf]

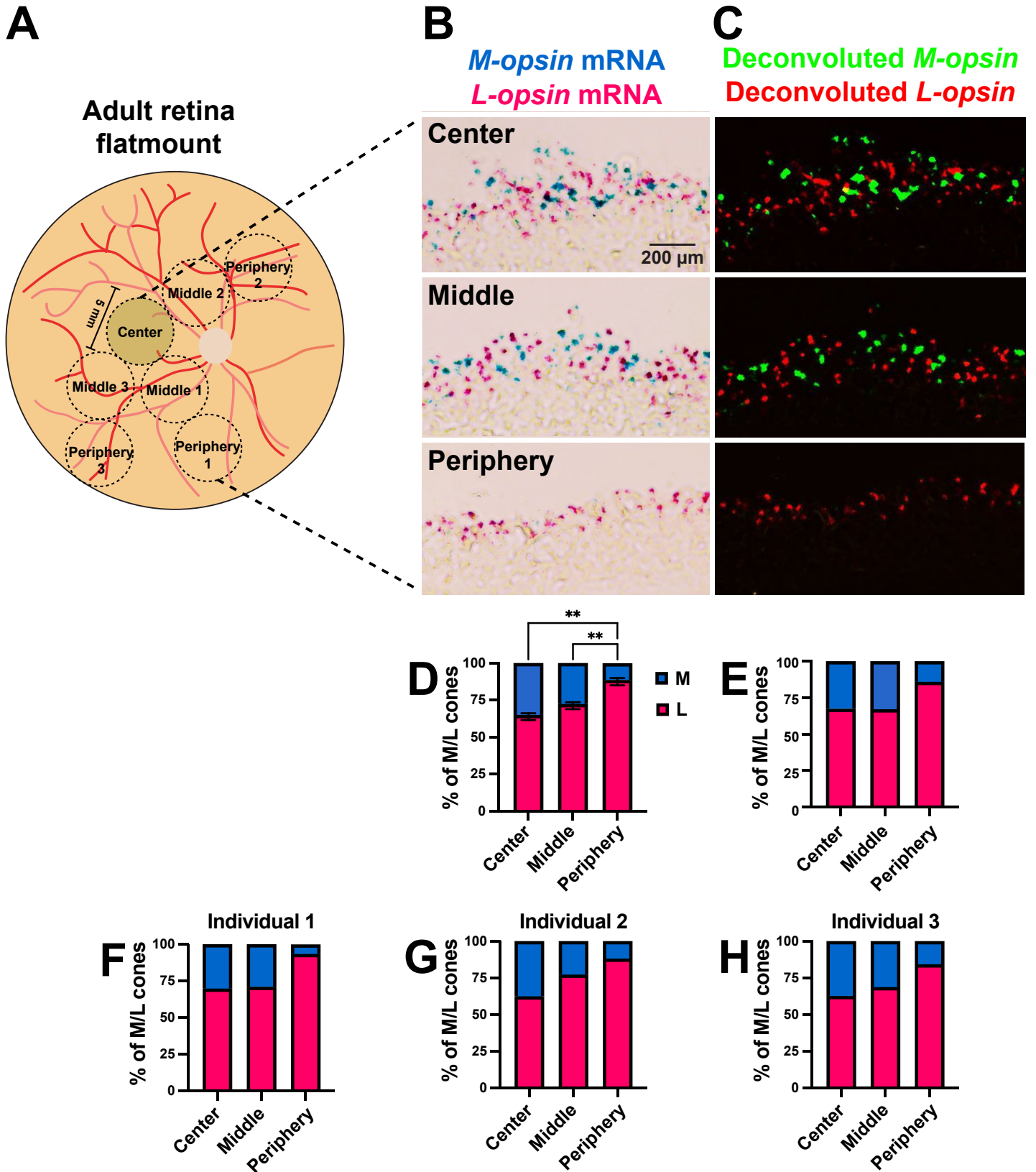

Supplemental Figure 3

Supplement: S3 Fig — (A) Schematic of retina with regions isolated using a 5 mm biopsy punch (as in Fig 2G with additional detail). White circle = optic nerve. Red lines = blood vessels. Yellow circle = macular pigment. (B–E) Validation of quantification of % of cells expressing M-opsin or L-opsin with HALO semi-automated image analysis software. (B) Images from 20 μm sections were probed for M-opsin (blue) and L-opsin (pink) mRNA. Images as in Fig 2H–2J. (C) HALO software deconvoluted the colorimetric M-opsin or L-opsin mRNA signals to generate pseudo fluorescent images (from Fig S3B). (D) Manually counted average ratios of M and L cones as percent of M/L total cones across 3 individuals. One-way ANOVA with Tukey’s multiple comparisons test: Center L versus Middle L = no significance; Center L versus Periphery L p < 0.01; Middle L versus Periphery L p < 0.01; ** indicates p < 0.01. Data as in Fig 2K for comparison to Fig S3E. (E) HALO semi-automated software analysis of single deconvoluted representative images from the center, middle, and periphery regions (Fig S3C), showed similar ratios of M and L cones as manually scored retinas (Fig 2K). (F–H) Ratios of M and L cones as percent of M/L total cones for each individual; n > 850 cones for each region for each individual. Averages ratios are shown in Fig 2K. (PDF) [file pbio.3002464.s003.pdf]

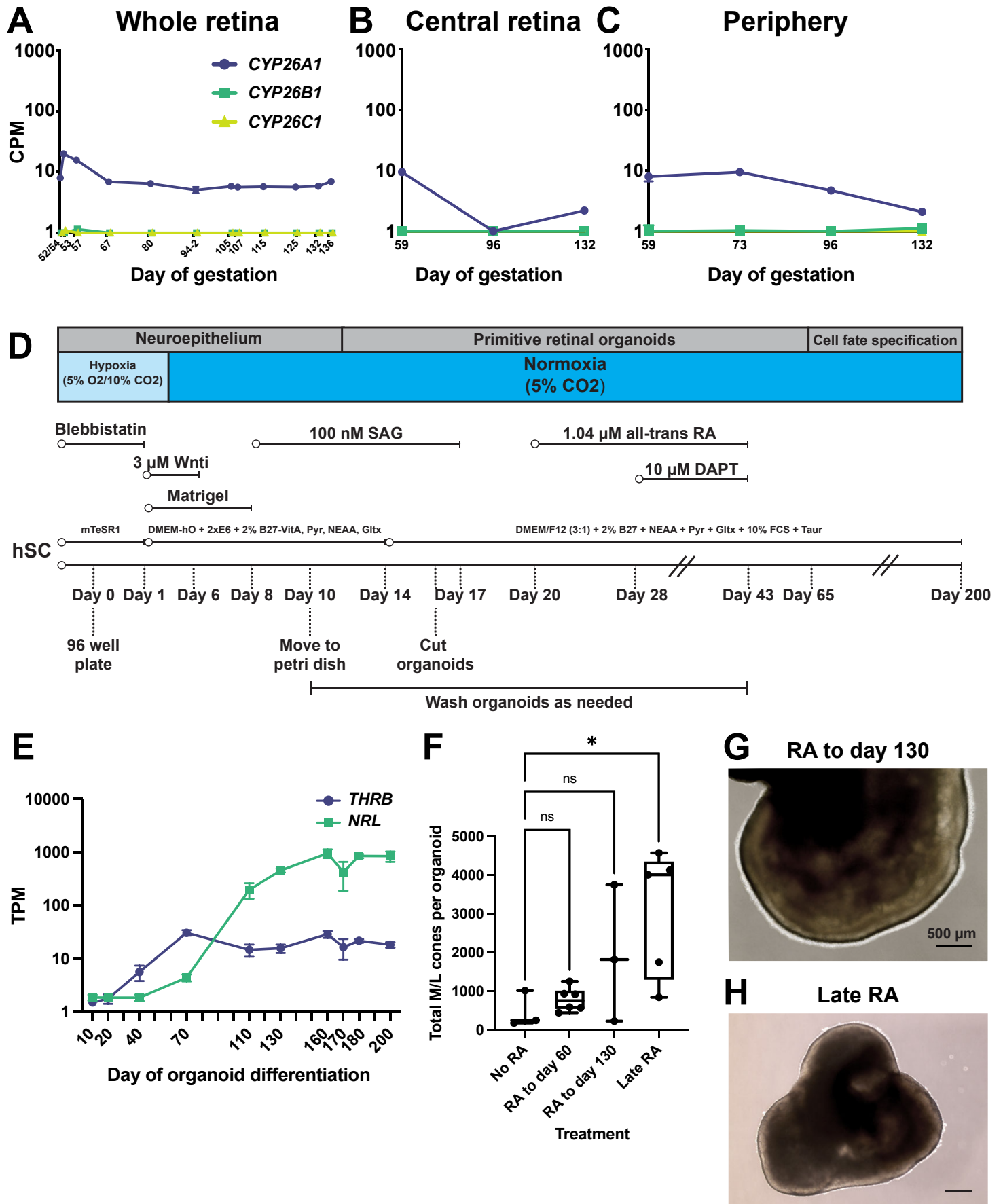

Supplemental Figure 4

Supplement: S4 Fig — (A–C) Expression of CYP26A1, CYP26B1, and CYP26C1 in fetal human retinas by day of gestation and retinal region. CPM, log counts per million. Analyzed from [16]. Error bars for the 2 samples from fetal day 94 indicate SEM. Original data sets are in S3 Data. (A) Whole retina. (B) Central retina. (C) Periphery. (D) Protocol for human retinal organoid differentiation, adapted from [15]. (E) Expression of THRB (cone marker) and NRL (rod marker) during retinal organoid development. TPM, transcripts per million. Analyzed from [15]. Original data sets are in S6 Data. (F) No significant differences in overall densities of M + L cones at day 200 in early RA treatment conditions (as in Fig 3F–3H) (Dunnett’s multiple comparison’s test, against “No RA” control: “RA to day 60” p = 0.98, “RA to day 130” p = 0.32). Significant difference between “No RA” and “Late RA” conditions (Dunnett’s multiple comparison’s test, * indicates p < 0.05) (as in Fig 3I). Error bars indicate SEM. Individual circles represent individual organoids. Original data sets are in S3 Data. (G) Representative brightfield image of a retinal organoid in “RA to day 130” conditions. (H) Representative brightfield image of a retinal organoid in “Late RA” conditions. (PDF) [file pbio.3002464.s004.pdf]

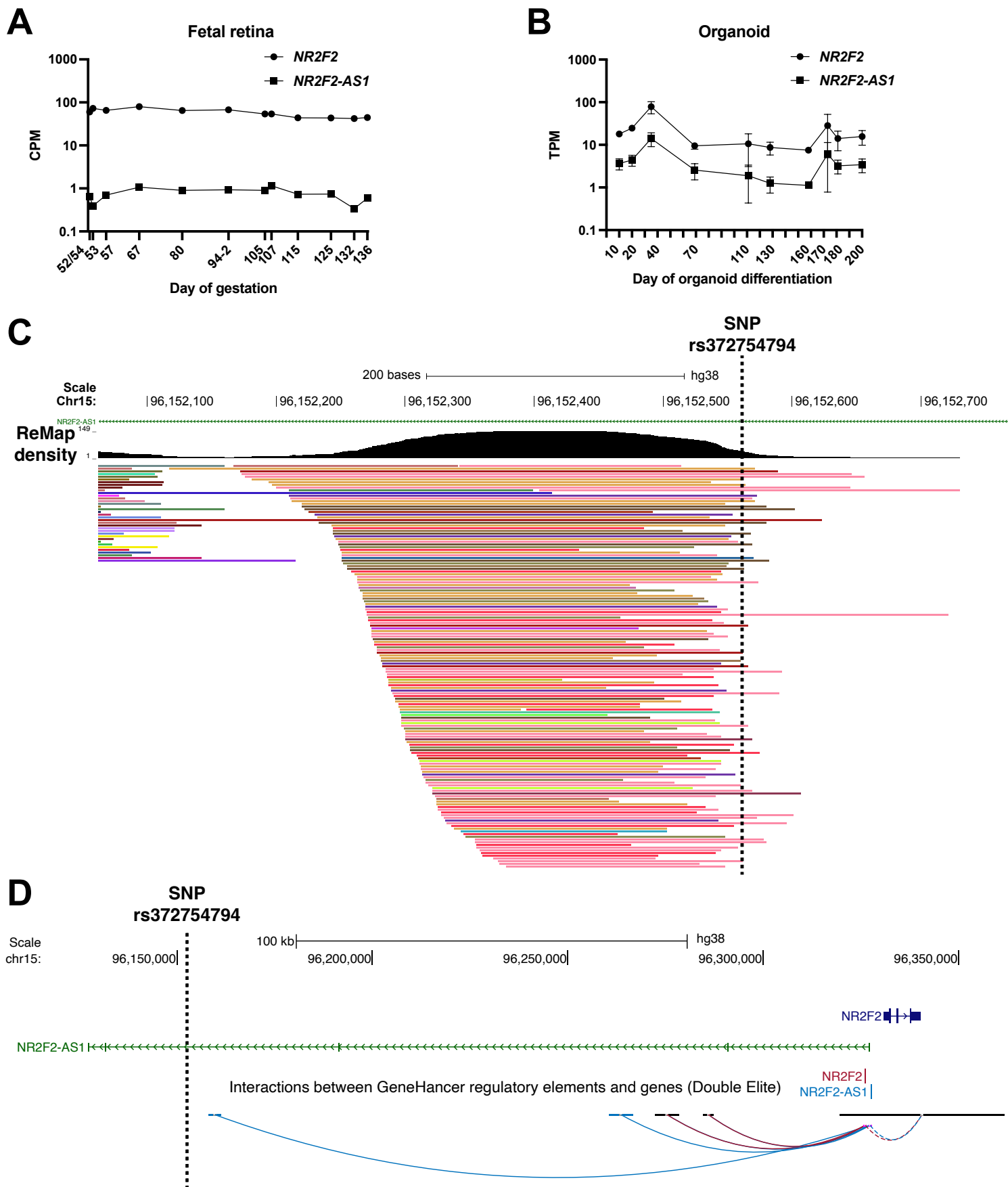

**Supplemental Figure 6**

Supplement: S6 Fig — The rs372754794 SNP at the NR2F2/NR2F2-AS1 locus lies in a putative regulatory region. (A) Expression of NR2F2 and NR2F2-AS1 in human fetal retinas, analyzed from [16]. Original data sets are in S3 Data. (B) Expression of NR2F2 and NR2F2-AS1 in human retinal organoids, analyzed from [15]. Original data sets are in S6 Data. (C) ReMap ChIP-seq database [52] shows that the rs372754794 SNP lies in an enhancer based on transcription factor binding. Each colored line indicates ChIP-seq binding data for a different transcriptional regulator. The ReMap density shows the density of the peaks overlap. (D) GeneHancer database [53] shows that the rs372754794 SNP neighbors a region predicted to physically interact and regulate NR2F2 and/or NR2F2-AS1. (PDF) [file pbio.3002464.s006.pdf]

**A**

**RARA rs36102671**

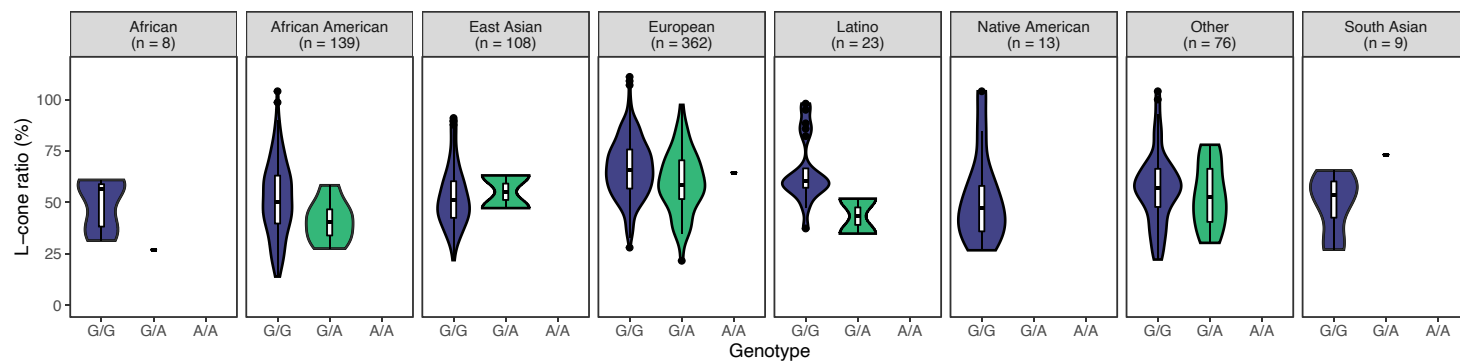

**B**

**RARA  
Whole Blood**

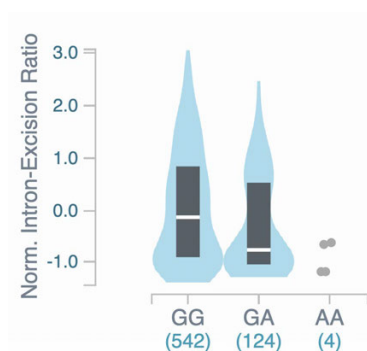

Supplement: S7 Fig — (A) Association of L:M ratio and rs36102671 stratified by self-reported ancestry. Original data sets are in S7 Data. (B) rs36102671 association with altered splicing of RARA in whole blood from the Genotype Tissue Expression Project (GTEx). Original data sets are in S7 Data. (PDF) [file pbio.3002464.s007.pdf]
