# Supplementary material for: Retinoic acid signaling regulates spatiotemporal specification of human green and red cones
Source: PLoS Biol. 2024 Jan 11;22(1):e3002464. doi: 10.1371/journal.pbio.3002464 (PMC10783767; doi:10.1371/journal.pbio.3002464)
Supplement: S5 Fig — Gene names and corresponding chromosome coordinates for human genome assembly GRCh38. (PDF) [file pbio.3002464.s005.pdf]

List of genes in association study and gene coordinates

Gene of Interest	chr#	Start	End
DIO1	chr1	53879779	53926574
PIAS3	chr1	145832505	145876560
RXRG	chr1	165346715	165499575
OTX1	chr2	63035597	63131383
NEUROD1	chr2	181647107	181693113
THRB	chr3	23970618	24697919
NR2F1 (COUP-TFI)	chr5	93319359	93628069
OPN1SW	chr7	128759936	128801444
RORB	chr9	74405551	74732495
CYP26A1/C1	chr10	93049490	93167890
NRL	chr14	24068098	24141275
OTX2	chr14	56755757	56970833
DIO2	chr14	80137530	80506478
DIO3	chr14	101447117	101623452
ONECUT1	chr15	52686989	52880012
NR2E3	chr15	71773279	71832289
NR2F2 (COUP-TFII)	chr15	96074584	96352953
RARA	chr17	40294657	40370655
ONECUT 2	chr18	57358933	57558283
SALL3	chr18	78908993	79079394
LW/MW gene region	chrX	154087755	154305671

Supplemental Figure 5
