# Supplementary material for: Retinoic acid signaling regulates spatiotemporal specification of human green and red cones
Source: PLoS Biol. 2024 Jan 11;22(1):e3002464. doi: 10.1371/journal.pbio.3002464 (PMC10783767; doi:10.1371/journal.pbio.3002464)
Supplement: Attachment — Submitted filename: 090923 Plos bio reviews REBUTTAL red green.docx [file pbio.3002464.s015.docx]

**Response to academic editor and reviewers**

We thank the academic editor and reviewers for their time, insight, and thoughtful input, which has enabled us to improve our manuscript. We addressed the suggestions, and our revised manuscript is now ready for publication in *PLoS Biology.*

We addressed the reviewers’ comments in the following manner:

1. We edited the text and figures to improve clarity and help the reader.
2. We conducted additional experiments to analyze M and L cones in fetal human retinas.
3. We validated our quantification of colorimetric *in situ* hybridization experiments using semi-automated computer software.
4. We expanded our analysis of publicly available genomics datasets.
5. We extended our explanation of the association study to clarify our experiments for non-experts. We explained how our association studies have the power to make our conclusions.
6. We edited our figures to provide zoomed out images of retinal sections and identified cell boundaries and retinal layers.
7. We provided information about how regionalization of expression reflects the development of the human retina from the center to the periphery.
8. We described how the irregular shapes of organoids could contribute to observations of dispersed cones in organoid sections.

We provide a point–by–point response below. Reviewers’ comments are in bold, and our responses to each comment are not in bold. Text from our manuscript is italicized.

**COMMENTS FROM THE ACADEMIC EDITOR:**

**The data presentation in the figures can be improved to help the reader. For example, it might be worth settling on a single quantification style for graphs like Fig. 2B-E, J, 3C,I,K S1B, S2D - these might be more easily interpreted if presented as boxplots?**

We appreciate the feedback regarding the style of the graphs. As the data in **Fig. 2C-F** (previous **2B-E**) and **S2B** (previous **S1B**) are pileup counts for individual reads from sequencing data, these values should be presented as individual points. We highlight this text explaining the data representation here:

*“We next quantified M- and L-opsin expression in RNA-seq datasets by comparing counts of aligned sequencing reads for M- or L-opsin based on 20 nucleotide differences that distinguish these genes (****Fig. 1A****), normalizing by the total number of aligned reads per sample (“normalized pileup count”). For these experiments, each point represents the normalized pileup count for reads with one nucleotide difference for M-opsin mRNA or L-opsin mRNA in that individual sample.”*

We now use box plots to make the data more easily interpretable for the cone densities in **S4F** (previous **S2D**). For **Fig.** **2K** (previous **2J**)**, 3E** (previous **3C**), **4B** (previous **3I**), and **4D** (previous **3K**), we retained the graph formats as we think that they convey the data clearly. **Fig.** **2K** (previous **2J**) and **3E** (previous **3C**) show quantification of proportions of cell types, which are well represented by bar graphs. **Fig.** **4B** (previous **3I**) is a box plot overlaid with a violin plot, providing a clear representation of the data. **Fig. 4D** (previous **3K**) is a Manhattan plot, presented in the standard format in the field.

**Graphs 3A, S2A,C should be made consistent, and include sensible error metrics;**

We unified the styles of **Fig. 3A**, **S4A** (previous **S2A**), and **S4E** (previous **S2C**) with regard to line and text size.

For **Fig.** **S4E** (previous **S2C**), we widened the error bars to make them easier to interpret.

**Fig. 3A** and **S4A** (previous **S2A**) are summaries of single timepoints in human fetal development (except in “94-2”, which has two data points). As such, these graphs display trends in gene expression changes. We clarify the data with the following text:

*“We assessed expression of RA pathway regulatory genes in 12 prenatal samples from day 52/54 through day 136 postconception from published RNA-seq datasets [16].”*

**Stylistically, it would also be helpful to use color throughout the figures in a more consistent fashion, with a focus on applying it where it can help understanding (ex M vs L). In some cases the color choices are a bit unhelpful - e.g. why are 3I,J green/orange?; why is 3A RGB?; why yellow in 3B, K doesn't need color at all, but if so, this particular color-code is a bit confusing - e.g. are we meant to perceptually group the blue-ish groups? I don't think this is the intention, but if it is, it might be better to plot them next to each other, seeing that the X axis is non-continuous anyway...**

We simplified or explained our color scheme to help clarify our results:

- **Fig. 4B,** **4C, 4G, 4H,** and **S7A** (previous **3I**, **3J, S4C, S4D**, **S9A**). We changed the graphs to colorblind friendly colors that are more muted (blue, green, yellow) to prevent confusion caused by the previous green/blue/orange color usage.
- **Fig. 3D** (previous **3B**). We changed the color coding to black for this figure.
- **Fig. 4D** (previous **3K**). For this Manhattan plot, each color represents variants in individual gene loci. The points represent the locations along the relative gene locus. *OPN1LW* and *OPN1MW* are two genes that are part of one locus. Due to the high similarity of their sequences, many reads could not be mapped, resulting in a gap in the variants. We clarify this with the following text:

*“****(D)*** *Manhattan plot of the genetic variant p-values. Dots represent genetic variants in genes. Genetic variants above the gray line (Bonferroni corrected threshold) are significant (p<0.05). The dots for each gene locus are presented in the same color (ex: DIO1 = dark blue; PIAS3 = orange). Along the X axis, the dots are spaced to scale as they occur in the genome. As OPN1LW and OPN1MW are nearby each other in the genome, the variants are represented together at the OPN1LW/MW locus in the same dark blue color. Due to the highly similar sequences of OPN1LW and OPN1MW, many reads could not be mapped, resulting in the gap in the variants at the OPN1LW/MW locus.”*

- **Fig. 3A, S4A** (previous **S2A**), **S4E** (previous **S2C**)**.** We changed the line graphs to color blind friendly colors that are more muted (blue, green, yellow) to prevent confusion caused by the previous red/green/blue color usage.

**Fig S1 could also be tightened up a bit, in terms of space**

For all supplemental figures, we tightened up the layouts.

**REVIEWS:
Reviewer #1: This is an elegant study that reports the prevalence of M cones over L cones during early human development. This was made possible thanks to the ingenious development of a highly sensitive probe that could differentiate between M and L-opsin mRNAs despite the high degree of similarity between them.
Using human stem cell derived retinal organoids, the paper also convincingly demonstrates that retinoic acid (RA) signalling is much more pronounced in early development and appears sufficient to explain the early prevalence of M cones and suppression of L cone fate. Experiments are well described and presented.**

We thank Reviewer 1 for their insightful and complimentary summary of our work.

**I have only one small issue with Figure 2B, showing M-opsin mRNA changes over post conceptional days. There seems to be a decrease between Day 132 and Day 136. Is that significant? and if so, does it indicate that marks the onset of downregulation in the production of M opsin mRNA at that age?**

This decrease is not likely significant. These analyses were conducted on single human fetal retinal samples, and thus this decrease likely was caused by biological variability. We updated the text to address this concern:

*“M-opsin expression increased in retinas from day 115 through 132. M-opsin expression was present but lower on day 136 (****Fig. 2C****). As these expression analyses were conducted on single human fetal retinal samples [**16**], the decrease is likely due to biological variability between samples.”*

**The second part of the paper describes variations in L cone prevalence in a human population sample from various ethnic backgrounds, showing that African and African American backgrounds are associated with a reduction in the natural ratio of L cones. This was found to be associated with a noncoding polymorphism in the NR2F2 gene which is associated with RA signalling, once again demonstrating a link between RA signalling and the promotion of M cones. I have no doubt about the validity of these findings. but that aspect of the paper is a little bit obscure to non-aficionados in the field of human genetics and would benefit from being presented in a slightly less jargonish style. The figures associated with that aspect of the study are really difficult to understand, with very little explanation in support.**

To clarify the association studies, we added text or highlighted text here that better describes the data in the figures:

**Fig. 4A** (previous **3H**)

*“We observed a range of L cone ratios (mean=59.1%; s.d.=16.9%;* ***Fig. 4A****). The association between FP-ERG and % L is nonlinear and as 100% is approached, very small changes in measured spectral sensitivities are associated with relatively large changes in estimates of % L, leading to some values that exceeded 100% upon normalization (****Fig. 4A****).”*

**Fig. 4B-C** (previous **3I-J**)

*“Individuals with the T/G genotype displayed a lower % L cone ratio than individuals with the T/T genotype (****Fig. 4B-C****).”*

**Fig. 4D** (previous **3K**)

*“We mapped the reads, genotyped the samples, and evaluated associations with L cone ratios (****Fig. 4D****). In this Manhattan plot (****Fig. 4D****), the position of each variant along the y-axis represents the significance of the association with the variation in L cone ratio and the position along the x-axis represents the genomic position at each locus.”*

**Fig. 4E-F** (previous **S4A-B**)

*“The SNP rs372754794, which occurs within the targeted sequence spanning NR2F2/COUP-TFII, lies in an intron in a non-coding RNA (NR2F2-AS1) immediately upstream of NR2F2 (****Fig. 4E-F****).”*

**Fig. 4F** (previous **S4B**)

*“Within African American populations from the 1000 Genomes Project, rs372754794 exhibited only modest linkage disequilibrium (LD) with other nearby variants (****Fig. 4F****), such that the associated haplotype is confined to the NR2F2-AS1 transcript.”*

**Fig. 4G** (previous **S4C**)

*“The minor (G) allele, which is associated with reduced L cone ratio, was nearly exclusive to African American and African subjects within our sample (MAF=0.0359;* ***Fig. 4G****) and exhibited similar patterns of frequency differentiation in external data from the 1000 Genomes Project [**40**].”*

**Fig. 4H** (previous **S4D**)

*“The association with L cone ratio was robust even when restricting analysis to African American subjects (*$\hat{\beta}$*=-14.45, p-value=1.44 × 10^-4^) (****Fig. 4H****).”*

**Fig. S6A-B** (previous **S5A-B**)

*“NR2F2 is a nuclear receptor that mediates RA signaling [**41**]. NR2F2 and the upstream NR2F2-AS1 are expressed in human retinas and retinal organoids during development (****Fig. S6A-B****)* *[**16**].”*

**Fig. S6C-D** (previous **S5A-B**)

*“The associated SNP lies in a region of high transcription factor binding identified as a putative enhancer (****Fig. S6C****) that is predicted to interact and regulate the promoters of NR2F2 and/or NR2F2-AS1* *(****Fig. S6D****).”*

**Fig. S7A** (previous **S6A**)

*“The minor (A) allele, which segregates at low allele frequencies in globally diverse populations, is associated with decreased L cone ratios (*$\hat{\beta}$*=-15.95, p-value=6.54 × 10^-5^) (****Fig. S7A****).”*

**Fig. S7B** (previous **S6B**)

*“The SNP exhibits an association with altered splicing of RARA based on RNA sequencing of whole blood (****Fig. S7B****) [**42**], suggesting that the haplotype modulates RARA function.”*

**Reviewer #2: In this study Hadyniak and colleagues have devised a method to estimate the relative mRNA expression of the medium (M-) and long (L-) wavelength sensitive cone photopigments (opsins) in human retina and retinal organoids. They use this tool to address an important question of when and how cones select between M - or L- subtype fates. Given the similarity in the protein sequence of M and L opsins, the authors designed unique nucleotide sequences that can specifically identify M- vs L-opsin. Using these probes the authors performed in situ hybridization to determine the expression pattern of M- and L-opsin in adult human retina and at different stages of fetal development. They validate this approach first in HEK cells transfected with M-opsin and L-opsin plasmid DNA followed by immunohistochemistry to confirm opsin protein expression. They next apply this method in post-mortem human retina and find that M-opsin expression precedes L-opsin expression with M-opsin mRNA starting to express ~ fetal day 115 and no L-opsin mRNA expression until midgestation. The authors also find that in adult human retina the ratio of L- to M-opsin expression varies across regions and attribute this to the temporal sequence of cone specification/development in central vs peripheral retina. To dissect the underlying mechanism of cone fate selection, the authors explore the role of retinoic acid (RA) pathway and find that not only is the expression of RA synthesizing enzymes high in early fetal retina but also that manipulating the duration of exposure to RA in the culture medium can alter the cone fate selection in human retinal organoids. Lastly, the authors address if the variability in the ratio of M and L cones previously reported in humans across population is related to RA signaling. Using a targeted sequencing approach the authors find a strong association of RA signaling related genes with differences in M and L cone ratios. Overall the study is interesting, the results are quite exciting, and paper is well-written. However, there are several concerns related to the experiments, results and figure presentation outlined below that need to be properly addressed before publication.**

We thank reviewer #2 for their thoughtful review and suggestions.

**1. I am concerned about the quality of the human retinal tissue as well as the labeling pattern that is being used to analyze/count M and L opsin expressing cones. For eg. it is difficult to understand or delineate from the images of human retina where is the opsin expression localized. One can't distinguish where the photoreceptor soma, inner and outer segments are in Fig 1K, 2A, G-I. It would be nice to have the entire retinal cross-section including a bright field image.**

For **Fig. 1L** (previous **1K**), we now provide an extended region of retina and circled photoreceptors. These data are the results of RNA in situ hybridization for *M-* and *L-opsin* RNA combined with immunohistochemistry for M/L protein (the antibody recognizes both M- and L-opsin protein). Due to the harsh treatment of this tissue (see Methods), the fragile outer segments often broke off. In this image, we observe M/L protein in all cells that contain *M-* and *L-opsin* RNA, though it appears that the outer segments have broken off. We determined cell boundaries by 1) identifying layers from a nuclear Hoechst stain (**Fig. S1B**), and 2) identifying IHC signal from the ONL to the OPL. We added this text to clarify our approach:

*“Cell boundaries were determined by identifying layers from a nuclear Hoechst stain (****Fig. S1B****) and analyzing opsin protein immunohistochemistry signal from the ONL to the OPL.”*

For **Fig. 2A**, we now show an extended region of the retinal tissue at fetal day 122 in which we only detect *M-opsin-*expressing cells and no *L-opsin-*expressing cells. We conducted an additional *in situ* hybridization on retinal tissue at fetal day 130, where we detected nearly all *M-opsin-*expressing cells with scarce *L-opsin* expressing cells (**Fig. 2A-B**). For this timepoint, we also imaged a large cross section of fetal tissue, which displayed opsin expression only in the central regions (**Fig. S2C-I**), consistent with the generation of cones starting central and progressing to the periphery. We quantified the proportion of M and L cones in the 122-day-old and 130-day-old human fetal retinas in **Fig. 2B**. Together, these data are consistent with our conclusion that *M-opsin* is expressed before *L-opsin* during human fetal development. We updated the text as follows:

*“To assess the timing of M and L cone specification during development, we evaluated M-opsin or L-opsin mRNA in a 122-day-old human fetal retina and a 130-day-old human fetal retina. We predicted two main, alternate outcomes: (1) a mix of cones expressing either M-opsin or L-opsin if the decision was stochastic, or (2) a majority of cones expressing M-opsin or L-opsin only if the decision was temporal and one cone subtype was generated first. In the 122-day-old retina, we observed M-opsin and no L-opsin (100% M-opsin only cells, n>500 cells) (****Fig. 2A-B****). This retina displayed M-/L-opsin protein in the ONL (****Fig. S2A****), suggesting that these cells were terminally differentiating M cones. This retina also displayed Ki67 expression (****Fig. S2A****), suggesting that there are proliferating cells, ongoing development, and generation of additional neurons at this timepoint in this region of the fetal retina. In the 130-day-old retina, we observed >99% M-opsin only cells with sparse L-opsin only cells (****Fig. 2A-B****). Opsin expression occurred within the outer nuclear layer (ONL,* ***Fig. S2I****) and spanned a ~3 mm wide central region of the retina (~38% of the total width of the globe), skewed towards the temporal side, between 1.6-4.5 mm from the most temporal edge (****Fig. S2C-H****). The highest number of M-opsin expressing cells were detected in the center of this range, approximately 3 mm from the temporal edge (****Fig. S2H****), consistent with observations indicating that the human retina develops from the center to the periphery [**2**]. Together, these data suggested that M cones are generated before L cones during human retinal development.”*

**For Figure 2A** and **2H-J (**previous **2G-I**), we added a line to indicate the edge of the retinas.

**Can these ISH labelings be done in whole mount retina preparation instead of sections? This will allow for better and more precise counting and estimation of M and L cones along with a better understanding of the cone mosaic.**

We agree that whole mount ISH labeling would provide a complementary strategy to assess *M-* and *L-opsin* RNA expression. Advanced Cell Diagnostics, the company that developed the BaseScope RNA *in situ* hybridization technology used for our experiments, does not have a protocol for whole mount tissue. We attempted ISH labeling in whole mount tissue preparations on several occasions, however, we only detected broad non-specific signal, despite testing numerous permutations of wash and incubation conditions. Though whole mount ISH labeling would nicely complement our data, our use of sectioned tissue enables us to compare the ratios of cone subtypes in tissue regions and draw conclusions about cone patterning in human retinal and organoids tissue.

**2. How do the authors explain the 2-fold decrease in M-opsin expression from fetal day 132 to 136 without any concomitant change in L-opsin expression?**

This decrease is not likely significant. These analyses were conducted on single human fetal retinal samples, and thus this decrease likely was caused by biological variability. We updated the text to address this concern:

*“M-opsin expression was observed from day 115 through day 136 (****Fig. 2C****). M-opsin expression increased in retinas from day 115 through 132. M-opsin expression was present but lower on day 136 (****Fig. 2C****). As these expression analyses were conducted on single human fetal retinal samples [**16**], the decrease is likely due to biological variability between samples.”*

**3. When estimating the M and L opsin expression in fetal retina especially >fetal day 110, which retinal location is being used to analyze? Previous studies have shown that at these fetal stages the fovea is starting to form and the central retina has a characteristic morphology with more cones packed in this region and expressing M/L opsin (Zhang et al Neuron 2020). Can the authors separately analyze central and peripheral retinal M vs L-opsin expression at these later timepoints?**

When estimating the *M-* and *L-opsin* expression in fetal retina in **Fig. 2C-F** (previous **2B-E**), we assessed expression in fetal retina RNA-seq data sets generated in Hoshino et al, which were conducted on whole retinas. Hoshino et al. conducted some analyses of central vs. periphery expression, however our computational pipeline detected no *M-* or *L-opsin* reads likely due to the minimal available tissue.

To address the reviewer’s comment with a different approach, we conducted an additional *in situ* hybridization on retinal tissue at fetal day 130, where we detected nearly all *M-opsin-*expressing cells with scarce *L-opsin* expressing cells (**Fig. 2A-B**). For this timepoint, we also imaged a large cross section of fetal tissue, which displayed opsin expression only in the central regions (**Fig. S2C-I**), consistent with the generation of cones starting centrally and progressing to the periphery.

**4. It will be important to mention which retinal location (in eccentricity or visual angle) are being referred to as center, middle and periphery in Fig 2F-J & Suppl Fig 1C-E. From the images it is not clear except there is some difference in cone density between periphery and the other two locations. How far is middle from periphery?**

We defined the regions relative to the center of the retina. We took 5 mm wide punches of the center, and immediately adjacent regions (i.e. middle and periphery). For each retina, we analyzed 1 center punch, 3 middle punches, and 3 peripheral punches. We updated **Fig. 2G** and **S3A** to indicate the 5 mm wide size of tissue isolation for each region.

We expanded our description of this approach in our updated text:

*“To assess cone distributions in the adult human retina, we dissected three retinas, used a biopsy punch to isolate 5 mm wide regions of tissue from the center, middle, and peripheral retina (****Fig. 2G, S3A****), sectioned the tissue, and performed our in situ hybridization strategy. For each retina, we analyzed one punch from the center region, three middle regions, and three peripheral regions (****Fig. S3A****).”*

In the **Materials and Methods** section in the **Supplemental Information**, we described the method with additional detail:

*“A 5 mm punch biopsy tool (RBP-50, Robbins Instruments) was placed over the macula based on the presence of macular pigment in combination with presence of an avascular region to remove the central retina. Punches were taken immediately adjacent for the middle retina, and immediately adjacent for the periphery. A total of 1 central punch, 3 middle punches, and 3 peripheral punches were taken from each eye (****Fig. S3A****).”*

**5. The authors find that peripheral human retina is largely dominated by L-opsin expression reaching as high as >90% in some cases. This is a striking observation and could potentially revise our understanding of colour processing in the peripheral human retina. In fact, the findings in this study is in contrast to previous functional measurements of cone mosaic in peripheral non-human primate retina (Field et al, Nature 2010) where the distribution of M and L cones is less skewed to one type. To validate the results, could the authors analyze multiple peripheral locations in both nasal and temporal retina?**

When conducting these experiments on adult human retinas, we took the biopsy punches and noted their positions relative to the center/middle/periphery. However, we did not note their positions relative to the nasal and temporal retina. As the main focus of this work is the specification of M and L cones relative to timing and the center-periphery axis, our analyses of adult retinal tissue address this goal.

**6. It will be important to demonstrate that the regional variation in the M- and L-opsin expression is also dependent on RA signaling by performing similar analysis as in Fig 3A, across different retinal locations.**

To address the reviewer’s comment, we evaluated the expression of RA regulatory genes by analyzing the datasets of fetal retinal regions generated in Hoshino et al. (**Fig. 3B-C** and **S4B-C**). *ALDH1A1* and *ALDH1A2* were expressed highest in the center on day 59 and decreased on days 96 and 132. *ALDH1A3* was not expressed in the center. In the periphery, *ALDH1A1* and *ALDH1A2* were expressed more highly than in the center on day 59, with decreases on days 73, 96, and 132. *ALDH1A3* was not expressed in the periphery. *CYP26A1* was steadily expressed in the center and periphery and *CYP26B1* and *CYP26C1* were not expressed. These data are consistent with (1) the overall decreases in *ALDH1A1* and *ALDH1A2* expression during human fetal retinal development and (2) high RA signaling early and decreasing during development.

We describe these analyses:

*“We next assessed spatiotemporal expression of RA pathway regulatory genes in samples taken from the center or periphery through fetal development from published RNA-seq datasets [**16**]. ALDH1A1 and ALDH1A2 were expressed highest in the center on day 59 and decreased on days 96 and 132 (****Fig. 3B****). ALDH1A3 was not expressed in the center (****Fig. 3B****). In the periphery, ALDH1A1 and ALDH1A2 were expressed highest on day 59 (****Fig. 3B-3C****), with decreases on days 73, 96, and 132 (****Fig. 3C****). ALDH1A3 was not expressed in the periphery (****Fig. 3C****). CYP26A1 was steadily expressed in the center and periphery and CYP26B1 and CYP26C1 were not expressed (****Fig. S4B-C****). Together, these data are consistent with (1) the overall decreases in ALDH1A1 and ALDH1A2 expression during human fetal retinal development (****Fig. 3A****), and (2) high RA signaling early and decreasing during development.”*

We also described our interpretation of the expression dynamics in the discussion:

*“ALDH1A1 and ALDH1A3 expression was observed in the central and peripheral retina on day 59 and decreased at later timepoints in both regions. ALDH1A1 and ALDH1A3 expression decreases to very low levels earlier in the central retina than periphery, reflecting the regionalized timing of retinal development, with the central region developing earlier than the periphery.”*

**7. I am a little puzzled about the overall expression pattern of M and L opsins in the retinal organoids. In figure panels 3D and E, the opsin expression is clumped or clustered in patches over the surface of the organoid, with large gaps in the middle even though in either case (panel D or E) the majority of the cones should express L or M opsin. Shouldn't the expression be more continuous across the organoid? This poses some basic questions such as what is the cone density in these organoids and also what is the ratio of rods to cones? The authors must use better pictures in general and overlay the images with bright field pictures so that it is clear what the readers are looking at.**

The irregular shapes of organoids can result in a mix of cross and tangential sections. To address the reviewer’s concern, we zoomed in on the images and added a line to show the edge of the organoid tissue for **Fig. 3F-I** (previous **3D-E**). We also added representative bright field pictures of organoids in **Fig. S4G-H**. We updated the text to clarify the information:

*“Based on M- and L-opsin expression, cones were identified near the surface of all organoids in the presumptive ONL (****Fig. 3F-I****). During dissection and sectioning, the irregular shapes of organoids (****Fig. S3G-H****) can result in a mix of cross and tangential sections, likely leading to the dispersed patterns of cones observed in some sections.”*

**8. As a control can the authors show that S opsin and rhodopsin expression i.e. the fraction of rods or S cones, in the retinal organoids are not impacted during the RA treatment.**

The editor noted: *“As a note, after discussion with the Academic Editor, we would not strictly require that you provide the data asked for in Reviewer 2's point 8 (the S opsin and rhodopsin expression analyses) - while interesting, we think this request may be beyond the scope of the current work.”*

We appreciate the suggestion from the reviewer. Nevertheless, we agree with the editor that these experiments are beyond the scope of the current work. We added text to describe what is known about the regulation of rods/Rhodopsin in human retinal organoids:

*“RA appears to play multiple roles in human photoreceptor specification. In addition to its role in M vs. L cone specification, RA regulates rod generation in human retinal organoids [**43-45**]. How RA coordinates the specification of cone and rod fate features is a key question for future studies.”*

**9. In this study the authors have estimated the fraction of cells expressing M or L opsin from the fluorescence signal derived from the ISH. Does the fluorescence signal vary from cell to cell and across preparation? Can the authors plot a distribution of the raw fluorescence intensities across cones in the human retina? How do the authors determine the threshold of expression level or fluorescence signal for cell selection/counting? Details about this should be incorporated in the methods.**

Expression was manually scored as on/off for *M-opsin* mRNA and *L-opsin* mRNA. We describe the quantification method in the **Materials and Methods** in the **Supplemental information:**

***“HEK293 in situ hybridization quantification***

*Images were tile scanned and all individual cells inside the region were manually counted using the Adobe photoshop count tool. Cells were delineated by presence of pink, blue, or purple signal with a boundary drawn around the signal. Images were contrasted to determine brightfield boundaries as needed. Non-expressing cells were not counted.*

***Human adult retina in situ hybridization quantification***

*For adult retinas, all serially sectioned punches of human retinas were imaged and counted manually and identified by presence of pink, blue, or purple signal using the Adobe photoshop count tool. Exclusive expression of M-opsin or L-opsin was observed in cones. Co-expression of M-opsin and L-opsin was never observed in human retinas (purple signal).”*

We validated this scoring strategy using a semi-automated image analysis approach.

*“We manually quantified cells expressing M-opsin mRNA or L-opsin mRNA and validated our approach using semi-automated image analysis software (****Fig. S3B-E****).”*

We updated the figures as follows:

***“Supplemental Fig. 3. Quantification of cells expressing M-opsin or L-opsin mRNA in adult retinas and validation***

***(A)*** *Schematic of retina with regions isolated using a 5 mm biopsy punch (as in* ***Fig. 2G*** *with additional detail). White circle=optic nerve. Red lines=blood vessels. Yellow circle = macular pigment.*

***(B-E)*** *Validation of quantification of % of cells expressing M-opsin or L-opsin with HALO semi-automated image analysis software.*

***(B)*** *Images from 20 μm sections were probed for M-opsin (blue) and L-opsin (pink) mRNA. Images as in* ***Fig. 2H-J****.*

***(C)*** *HALO software deconvoluted the colorimetric M-opsin or L-opsin mRNA signals to generate pseudo fluorescent images (from* ***Fig. S3B****).*

***(D)*** *Manually counted average ratios of M and L cones as percent of M/L total cones across three individuals. One-way ANOVA with Tukey’s multiple comparisons test: Center L versus Middle L=no significance; Center L versus Periphery L p<0.01; Middle L versus Periphery L p<0.01. ** indicates p<0.01. Data as in* ***Fig. 2K*** *for comparison to* ***Fig. S3E****.*

***(E)*** *HALO semi-automated software analysis of single deconvoluted representative images from the center, middle, and periphery regions (****Fig. S3C****), showed similar ratios of M and L cones as manually scored retinas (****Fig. 2K****).”*

A description of the methods for this semi-automated image analysis approach is provided in the **Materials and Methods** in the **Supplemental information:**

***“Semi-automated quantification of M- and L-opsin in adult retina***

*Representative pictures of M- and L-opsin in situ hybridization colorimetric staining from central, middle, and peripheral regions of the adult retina were deconvoluted in HALO Image Analysis Platform 3.5.3577 (Indica Labs, Inc.)[1]. To visualize the chromogenic stain, we used the Deconvolution v1.1.7 module of the HALO Image Analysis Platform (Indica Labs, Inc.) to separate out the colorimetric stains of the RGB images. Specifically, average RGB values from 9-16 pixels of each chromogenic stain were input into the brightfield algorithm to generate deconvoluted images. The chromogenic RED-A/B signal (generated by alkaline phosphatase against the BA-Hs-OPN1LW-1zz-st-C2 probe) was assigned red pseudo color and GREEN-A/B signal (generated by horseradish peroxidase against the BA-Hs-OPN1MW-1zz-st1 probe) was assigned green pseudo color. If retinal pigmented epithelium was present, this was assigned orange pseudo color. The deconvoluted staining was then used to count the number of M- and L-opsin-expressing foci using the Object Colocalization FL v2.1.4 module.”*

**Reviewer #3: The primary goal of the manuscript is to explore the mechanisms specifying M vs L cone subtype fate. The authors first designed specific probes to detect of the expression of highly similar M and L-opsin mRNA and examined the timing of M and L-opsin mRNA expression in human retina by in situ hybridization and by analysis of the existing RNA-seq datasets. The work suggests that M cones are generated before L cones. The study further adds that retinoid acid promotes M cone fate early in development to generate the pattern of M/L cones in human retina. Finally the authors evaluated natural variation in the ratios of M and L cones in human population and identified a SNP within NR2F2/COUP-TFII locus that is significantly associated with variation in the L cone ratio. They also noted a second but statistically non-significant association with a SNP in RARA gene. These findings are consistent with the role of RA signaling in specifying M and L cone subtypes in human retina.**

**Overall, the study presents some interesting findings. However, the manuscript seems to be stitched together by combining different experiments, which are not fully explored. The data are limited and somewhat incomplete to justify the conclusions.  The manuscript includes three main figures. Fig 1 primarily tests the specificity of probes. Fig 2 presents data from M- and L-opsin expression based on bioinformatic analysis of one published human fetal retina dataset and one small dataset of adult human retina. It must be mentioned that gene expression in humans shows extensive variations. It doesn't take long to check multiple other datasets that are published from fetal, organoid and adult retinas. In fact, published scRNA-seq data can also help resolve this issue.**

To address the reviewer’s comments, we first analyzed two published scRNA-seq datasets from the developing human fetal retina.

The first dataset (from Sridhar et al. *Cell Rep* 2020), spanned day 59-125 during human fetal development. With the exception of 2 cells expressing *M-opsin* RNA on day 105, no cells expressing *M-* or *L-opsin* RNA were identified in this dataset.

The second dataset (from Lu et al. *Dev Cell* 2020) assessed expression in human fetal and adult retinas and human retinal organoids. The human retinal dataset spanned gestational week (GW) 9-27, postnatal day 8, and adult. The human organoid dataset spanned days 24-59 of organoid development. Across all 17 GW datasets, only 19 cells expressed either *M-* or *L-opsin* out of 42,834 total cells. The postnatal day 8 dataset had 87 cells expressing either *M-* or *L-opsin*. The adult dataset contained cells that expressed *M-*/*L-opsin* but the reads were not alignable and could not be distinguished as distinct *M-* or *L-*opsin reads.

Below is a figure generated from the authors’ browser interface displaying the number of cells that express *M-* or *L-opsin* (shown in parentheses).

**Organoid**

**Adult retina**

**Fetal retina**

**Postnatal day 8**

Though the total number of cells is low, more cells express *M-opsin* than *L-opsin* at earlier timepoints during fetal development. At postnatal day 8, more cells express *L-opsin* than *M-opsin*.

To independently validate these assessments, we re-analyzed the data and generated UMAP representations of OPN1MW (*M-opsin*) (left) and OPN1LW (*L-opsin*) (right) expression in the later fetal datasets (Weeks 12-27).

Similar to the analysis provided by the authors’ browser interface, we detected more cells expressing *M-opsin* than *L-opsin* during fetal development.

Though sparse, these data are consistent with our other approaches showing that (1) *M-opsin* is expressed before *L-opsin* during fetal retinal development (**Fig. 2A-D**) and (2) more cells express *L-opsin* than *M-opsin* after development is completed (**Fig. 2H-J**). As these data are sparse, we are not including this analysis in the manuscript.

**In situ shown is from only one fetal day 122 retina. Previous immunohistochemistry studies by Hendrickson and others showed expression of S-opsin even before L/M in the central foveal region but later only L/M opsin is detected in fovea, suggesting that a detailed analysis (and not a single timepoint) is required to make specific conclusions. Therefore, in situ probes don't add much to the information that is already obtained from computational work.**

To address the reviewer’s comment, we conducted RNA *in situ* hybridization on a fetal day 130 retina. We detected nearly all *M-opsin-*expressing cells with scarce *L-opsin* expressing cells (**Fig. 2A-B**). For this timepoint, we also imaged a large cross section of fetal tissue, which displayed opsin expression only in the central regions (**Fig. S2C-I**), consistent with the generation of cones starting central and progressing to the periphery. We quantified the proportion of M and L cones in the 122-day-old and 130-day-old human fetal retinas in **Fig. 2B**. Together, these data are consistent with our conclusion that *M-opsin* is expressed before *L-opsin* during human fetal development. We updated the text as follows:

*“To assess the timing of M and L cone specification during development, we evaluated M-opsin or L-opsin mRNA in a 122-day-old human fetal retina and a 130-day-old human fetal retina. We predicted two main, alternate outcomes: (1) a mix of cones expressing either M-opsin or L-opsin if the decision was stochastic, or (2) a majority of cones expressing M-opsin or L-opsin only if the decision was temporal and one cone subtype was generated first. In the 122-day-old retina, we observed M-opsin and no L-opsin (100% M-opsin only cells, n>500 cells) (****Fig. 2A-B****). This retina displayed M-/L-opsin protein in the ONL (****Fig. S2A****), suggesting that these cells were terminally differentiating M cones. This retina also displayed Ki67 expression (****Fig. S2A****), suggesting that there are proliferating cells, ongoing development, and generation of additional neurons at this timepoint in this region of the fetal retina. In the 130-day-old retina, we observed >99% M-opsin only cells with sparse L-opsin only cells (****Fig. 2A-B****). Opsin expression occurred within the outer nuclear layer (ONL,* ***Fig. S2I****) and spanned a ~3 mm wide central region of the retina (~38% of the total width of the globe), skewed towards the temporal side, between 1.6-4.5 mm from the most temporal edge (****Fig. S2C-H****). The highest number of M-opsin expressing cells were detected in the center of this range, approximately 3 mm from the temporal edge (****Fig. S2H****), consistent with observations indicating that the human retina develops from the center to the periphery [**2**]. Together, these data suggested that M cones are generated before L cones during human retinal development.”*

**Fig 3 focuses on RA signaling. The role of thyroid hormone and RA signaling is well documented by many studies. RA addition in organoids is interesting. But, several papers have shown the involvement of RA signaling in rod differentiation in retinal organoids. The data being shown is less than optimal to make a conclusion.**

Our studies in this paper identify the first cell fate specification mechanism that regulates human M vs. L cone fates. In **Fig. 3E-F**, we show that organoids grown in the absence of exogenous RA are enriched for L cones. In **Fig. 3E, 3G-H,** we show that addition of RA early in development dramatically promotes M cones and suppress L cones. In **Fig. 3E, 3I**, we show that addition of RA late does not change the ratio relative to the no RA controls. Together, these data support our conclusion that addition of RA early in development is sufficient to promote M cones and suppress L cones.

With regard to the images, the irregular shapes of organoids can result in a mix of cross and tangential sections. To address the reviewer’s concern, we zoomed in on the images and added a line to show the edge of the organoid tissue for **Fig. 3F-I** (previous **3D-E**). We also added representative bright field pictures of organoids in **Fig. S4G-H**. We updated the text to clarify the information:

*“Based on M- and L-opsin expression, cones were identified near the surface of all organoids in the presumptive ONL (****Fig. 3F-I****). During dissection and sectioning, the irregular shapes of organoids (****Fig. S3G-H****) can result in a mix of cross and tangential sections, likely leading to the dispersed patterns of cones observed in some sections.”*

RA has been shown to regulate rod differentiation. We added text to discuss the multiple roles of RA in photoreceptor specification:

*“RA appears to play multiple roles in human photoreceptor specification. In addition to its role in M vs. L cone specification, RA regulates rod generation in human retinal organoids [**43-45**]. How RA coordinates the specification of cone and rod fate features is a key question for future studies.”*

**The human genetics part is a valuable addition but lacks power and is shown in the supplement. The selection of SNPs also biases the association analysis. These studies are potentially very interesting but require a larger cohort and independent replication.**

Prior to this study, no genes or pathways had been identified that regulate human M vs. L cone fate. We used a candidate approach to measure association of variants in genes and pathways involved in vertebrate retinal development with % L cone fate. The results of our approach associated variants in RA regulatory genes with this fate decision. Our study has the statistical power to make this conclusion. The SNP rs372754794 was associated with a p-value=1.67 × 10^-5^, achieving statistical significance upon multiple testing correction (Bonferroni correction p-value threshold=1.73 × 10-5).

As these experiments were conducted in parallel with our other studies, these results independently implicated RA signaling in the M vs. L cone fate decision. We agree that a genome-wide study with a larger cohort could lead to the identification of additional variants in other genes, providing an interesting future direction. We highlight this text, in which we discuss how the results of the association study are consistent with the results of our other approaches:

*“Together, the association of RA-related candidates with changes in M and L cone ratios is consistent with a role for RA signaling in the specification of M and L cone subtypes in humans.”*

*“RA-synthesizing ALDHs are highly expressed early in retinal development and decrease over time, suggesting that RA signaling is high during early development. RA is sufficient to promote M cone fate and suppress L cone fate in immature cones.”*

We agree with the reviewer that we should move some data from the supplement to the main figure to improve data accessibility and interpretation. We made a new figure (**Fig. 4**), which displays the association study data, the effects of SNP rs372754794, the position of SNP rs372754794 in the genome, the local LD plot, and the association of SNP rs372754794 stratified by ancestry.

**Additional comments:**

**1.      The statement that "trichromacy is unique to primates among mammals" is incorrect.  Some Marsupials are trichromats as well, see a few PMID: 24737644, 21151905, 16859843, 11967153**

Thank you for bringing this inaccurate text to our attention. We updated the text to:

*“Trichromacy is unique to primates among placental mammals, enabled by blue (short/S), green (medium/M), and red (long/L) cones.”*

**2.      Please provide another image for Fig .1 H, that correlates with panel C and I of Fig.1.**

The experiment in **Fig. 1H** was an RNA *in situ* hybridization, whereas the experiment in **Fig. 1I** was an RNA *in situ* hybridization together with immunohistochemistry/IHC (see supplemental Materials and Methods). As such, the differences in color are due to the differences in the protocols, particularly the additional steps in the combined RNA *in situ* hybridization and IHC experiment that led to some reduction in signal.

We added text to clarify these differences:

*“****(I)*** *Visualization of M-opsin mRNA, L-opsin mRNA, and M-/L- opsin protein (black) in HEK293 cells transfected with both a plasmid driving M-opsin and a plasmid driving L-opsin. M-opsin (blue) and L-opsin (pink). Blue arrow indicates a cell expressing M-opsin mRNA only. Pink arrows indicate cells expressing L-opsin mRNA only. Purple arrow indicates a cell expressing both M-opsin mRNA and L-opsin mRNA. Black arrow indicates an untransfected cell. Cells were identified based on nuclear Hoechst staining (****Fig. S1A****). With this combined RNA in situ hybridization/immunohistochemistry approach, the mRNA signal was reduced, when compared to the mRNA signal observed when RNA in situ hybridization was conducted alone (****Fig. 1H****).”*

**3.      In Figure 1K, a low magnification image of the retina section should be included as well.**

For **Figure 1K**, we now provide an image of an extended region of the retina and circled photoreceptors. These data are the results of RNA in situ hybridization for *M-* and *L-opsin* RNA combined with immunohistochemistry for M/L protein (the antibody recognizes both M- and L-opsin protein). Due to the harsh treatment of this tissue (see Methods), the fragile outer segments often broke off. In this image, we observe M/L protein in all cells that contain *M-* and *L-opsin* RNA, though it appears that the outer segments have broken off. We determined cell boundaries by 1) identifying layers from a nuclear Hoechst stain, and 2) identifying IHC signal from the ONL to the OPL. We describe these data and approaches with the following text:

*“We next tested this assay in adult human retinas. We identified cells that exclusively expressed M-opsin or L-opsin (****Fig. 1L****). M-/L-opsin protein was observed in nearly all cells that expressed M-opsin or L-opsin mRNA (****Fig. 1K-L, S1B-C****), suggesting that this method identifies opsin protein-expressing M- and L-cones.”*

*“****(L)*** *Visualization of M-opsin mRNA, L-opsin mRNA, and M-/L- opsin protein in cone cells in an adult human retina. M-opsin (blue) and L-opsin (pink). No cones co-expressed M-opsin mRNA and L-opsin mRNA. ONL = outer nuclear layer; OPL = outer plexiform layer; INL = inner nuclear layer. Cell boundaries were determined by identifying layers from a nuclear Hoechst stain (****Fig. S1B****) and analyzing opsin protein immunohistochemistry signal from the ONL to the OPL.”*

*“****(B)*** *Identification of retinal layers using Hoechst (light gray) in the experiment in* ***Figure 1K-L****. M-opsin (blue) and L-opsin (pink). No cones co-expressed M-opsin mRNA and L-opsin mRNA. ONL = outer nuclear layer; OPL = outer plexiform layer; INL = inner nuclear layer.*

**In Supplemental Figure 1A, DAPI staining should be performed to highlight the retinal layers.**

For **Fig S2A** (previous **S1A**), we added an image with Hoechst staining to highlight the retinal layers.

**4.      Fig.1I lacks negative control. In Fig.1 I-L How many cells were taken? Be specific with the number of cells counted. Provide a table in the supplementary about the accuracy/confidence of the method**

We provide a table in the supplement (**Fig. S1C**) in which we provide the numbers of opsin mRNA expressing cells that express opsin protein. We describe this subfigure here:

***“(C)*** *Quantification of M-opsin mRNA-expressing, L-opsin mRNA-expressing, or M+L-opsin mRNA-expressing cells that express M/L-opsin protein for the experiments conducted in HEK293 cells in* ***Fig. 1I-J*** *and adult human retina in* ***Fig. 1K-L****.”*

For **Fig. 1I**, we now indicate an untransfected cell in the field. We describe this update to the figure with this text:

***“(I)*** *Visualization of M-opsin mRNA, L-opsin mRNA, and M-/L- opsin protein (black) in HEK293 cells transfected with both a plasmid driving M-opsin and a plasmid driving L-opsin. M-opsin (blue) and L-opsin (pink). Blue arrow indicates a cell expressing M-opsin mRNA only. Pink arrows indicate cells expressing L-opsin mRNA only. Purple arrow indicates a cell expressing both M-opsin mRNA and L-opsin mRNA. Black arrow indicates an untransfected cell. Cells were identified based on nuclear Hoechst staining (****Fig. S1A****). With this combined RNA in situ hybridization/immunohistochemistry approach, the mRNA signal was reduced, when compared to the mRNA signal observed when RNA in situ hybridization was conducted alone (****Fig. 1H****).”*

**5.      Fig. 2A lacks controls. Another image with defined cell boundaries must be included.**

We updated this image to show a broader cross section, identified cell boundaries with circles, and identified the edge of the retina with a line. We also analyzed an additional retina at fetal day 130 (**Fig. 2A-B, S2C-I**).

**6.      The authors should explain why they examined RA signaling.**

We edited the text to explain why we examined RA signaling:

*“We next sought to identify a mechanism that regulates M and L cone specification. RA signaling controls expression of an independently evolved opsin gene array in zebrafish [**20-22**]. We hypothesized that RA may play a role in the regulation of the M/L-opsin gene array in the developing human retina.”*

**In relation to this question, in Figure 3C, what causes the high proportion of L cone in "No RA" condition?**

The high proportion of L cones in “no RA” are a result of the control organoid differentiation strategy. The 87% L cone ratio is similar to the proportions that we observe in our evaluation of adult human tissue, consistent with our and others’ previous studies suggesting that human retinal organoids recapitulate normal human retinal development and cell fate specification.

We highlight this similarity with the following text:

*“These organoids displayed a mix of M cones and L cones (****Fig. 3E-F****), similar to adult human retinas (****Fig. 2H-K****).”*

**Also, the bar graph in Figure 3C does not show the purple "M+L" as indicated on the right.**

The population of M+L opsin expressing cells in our organoids experiments was extremely small. To clarify this for the reader, we now describe these proportions in the text:

*Organoids were grown in media without exogenous RA from day 43 to day 200, from the end of primitive retina differentiation through early cell fate specification (13% M, 87% L, 0.1% co-expressing M+L;* ***Fig. 3D-F,*** ***‘No RA’****).*

*“Addition of RA early through cone maturation from days 43 to 130 yielded organoids with almost exclusively M cones at day 200 (98% M, 2% L, 0% co-expressing M+L;* ***Fig. 3E, 3H****;* ***‘RA to day 130’****), suggesting that addition of RA through the beginning of M- and L-opsin expression was sufficient to promote M cone fate.”*

*“To determine whether RA could promote M cone fate in immature cones, we added RA through day 60, before the end of immature cone generation and well before terminal differentiation and M- and L-opsin expression on day 130. In this condition, organoids were enriched for M cones (74% M, 26% L, 0% co-expressing M+L;* ***Fig. 3E, 3G;*** *‘****RA to day 60****’), suggesting that RA was sufficient to promote M cone fate in immature cones.”*

*“Addition of RA late in development during terminal differentiation from days 130 to 200 yielded L cone-enriched organoids at day 200 (6% M, 93% L, 1% co-expressing;* ***Fig. 3E, 3I; ‘Late RA’****), similar to ‘No RA’ control organoids (****Fig.*** ***3E-F****), suggesting that RA was not sufficient to convert L cones to M cone fate.”*

**7.      Fig. 3G-D : include bright field image of retinal organoids.**

When conducting these experiments, we took bright field images of organoids in the “RA to day 130” and “Late RA” conditions, which we added in **Fig. S4G-H**.

**8.      Is the effect of RA dose-dependent? Did the authors examine the effect of lower or higher concentrations of RA in organoids?**

We did not examine the dose-dependence of RA. We used 1.0 μM RA as this concentration is part of the differentiation phase of organoid development in our control experiments (**Fig. S4D**). We highlight the following text, explaining this rationale:

*“We tested this hypothesis using human retinal organoids. Differentiation of human retinal organoids involves addition of 1.0 µM RA on days 20-43 to promote early retinal patterning (****Fig. S4D****). Organoids were grown in media without exogenous RA from day 43 to day 200, from the end of primitive retina differentiation through early cell fate specification (13% M, 87% L, 0.1% co-expressing M+L;* ***Fig. 3D-F,*** ***‘No RA’****). These organoids displayed a mix of M cones and L cones (****Fig. 3E-F****), similar to adult human retinas (****Fig. 2H-K****).*

*To test whether RA was sufficient to promote M cone specification in retinal organoids, we added 1.0 μM RA over different timeframes and assessed M and L cone ratios at day 200 (****Fig. 3D****).”*

**9.      The authors states that "addition of RA late in organoid development likely improves cone survival but not fate specification", but also that "organoids grown in supplemental RA throughout development failed to normally differentiate yielding minimal to no M or L cones". Can the authors explain the discrepancy? Did the organoids die in supplemental RA throughout development? Why did it repress differentiation?**

When RA was added to the media continuously from days 43-200, the organoids failed to differentiate proper outer segments and healthy photoreceptors. When RA was added early (before day 130), organoids were enriched for M cones, with no change in final cone density. When RA was added late (after days 130), it did not change their fate (No RA versus Late RA), but did yield an increase in the final cone density. We conclude that high continuous RA is either detrimental to cone health in general or specifically for M cone survival. We added text to describe this:

*“Addition of RA throughout development prevented normal development and observation of M or L cones at day 200, suggesting that continuous high RA is detrimental to overall cone or organoid health. Alternatively, as high RA early (****Fig 3E, 3H****) yields organoids enriched for M cones, late RA may specifically impair M cone survival.”*

**10.     The authors defined three main timeframe of cone development: immature cone generation, maturation, and terminal differentiation. Terminal differentiation usually includes morphogenesis, e.g., formation of outer segment, and the acquisition of phototransduction ability. How does it differ from "maturation", in terms of cellular and molecular changes?**

We define (1) generation as the time when cones are generated, as indicated by THRB expression, (2) maturation as the time between initial generation and terminal differentiation, and (3) terminal differentiation as the onset of expression of terminal genes including opsins. We highlight this text that describes how we came to these terms:

*“We previously examined gene expression at multiple timepoints during organoid development using bulk RNA-seq [**15**]. We analyzed this data and found that expression of thyroid hormone receptor beta (THRB), a marker of cone fate, stabilizes on day 70 (****Fig. S4E),*** *suggesting that the generation of new cones ends around this timepoint. In contrast, expression of neural retina leucine zipper (NRL), a marker of later-born rod photoreceptors, stabilizes around day 160 (****Fig. S4E****). Opsin expression was first observed at day 130 [**15**], suggesting that terminal differentiation of cones begins at this timepoint. We defined three main timeframes of cone development: immature cone generation through day 70, maturation from days 70 to 130, and terminal differentiation from day 130 onwards.”*

**11.     Do human retinal organoids also display higher proportion of M cones in the center, as in human retina?**

In our studies, we find that organoids do not have a central fovea/macular-like structure as seen in human retinas. Work from Susanna Da Silva has suggested some regional differences. However, due to the nature of the organoid tissue, it has not yet been shown whether these observations are truly the generation of central retina features or variation in this heterogenous tissue. In our work, we consider the organoid as a whole when describing cone proportions.
